# Bridging Research and Clinical Practice: Automated [^68^Ga]Ga-FAPi-46 Synthesis and Quality Control for Oncological PET Imaging

**DOI:** 10.3390/ph19040594

**Published:** 2026-04-08

**Authors:** Caiubi Rodrigues de Paula Santos, Luciana Malavolta, Jorge Mejia, Leonardo Lima Fuscaldi, Lilian Yuri Itaya Yamaga, Marycel Figols de Barboza

**Affiliations:** 1 Einstein—Hospital Israelita, Sao Paulo 05652-900, Brazil; luciana.malavolta@gmail.com (L.M.); jorge.mecabeza@einstein.br (J.M.); lilian.yamaga@einstein.br (L.Y.I.Y.); marycel.barboza@einstein.br (M.F.d.B.); 2 Department of Physiological Sciences, School of Medical Sciences, Santa Casa de Sao Paulo, Sao Paulo 01221-020, Brazil; leonardo.fuscaldi@hotmail.com

**Keywords:** [^68^Ga]Ga-FAPi-46, gallium-68, fibroblast activation protein inhibitor, automated synthesis, PET imaging

## Abstract

**Background/Objectives:** Fibroblast activation protein (FAP) has emerged as a promising target for oncologic molecular imaging due to its high expression in cancer-associated fibroblasts and low presence in healthy tissues. Among available FAP ligands, [^68^Ga]Ga-FAPi-46 has shown rapid tumor accumulation, low background uptake, and broad tumor applicability. This study reports the successful translation of [^68^Ga]Ga-FAPi-46 from preclinical development to routine clinical radiopharmacy practice, detailing automated synthesis, quality control performance, radiochemical stability, and the first clinical imaging results. **Methods:** Automated radiolabeling of FAPi-46 with gallium-68 was performed using a synthesis module. Quality control included radiochemical purity assessments by iTLC, SPE, and RP-HPLC (pH, appearance, endotoxin levels, and membrane integrity testing). Radiochemical stability was evaluated in saline (up to 6 h) and human serum (up to 90 min). In vitro characterization included the partition coefficient and serum protein binding determination. A clinical evaluation was conducted in a woman with newly diagnosed lung adenocarcinoma who underwent both [^18^F]FDG PET/CT and [^68^Ga]Ga-FAPi-46 PET/CT. **Results:** Automated synthesis of [^68^Ga]Ga-FAPi-46 achieved a high radiochemical yield (87.9 ± 1.3%) and radiochemical purity greater than 98%. All batches met release specifications for sterility, apyrogenicity, and physicochemical parameters. The radiotracer demonstrated high stability in saline and human serum, with radiochemical purity consistently above 95% at all evaluated time points. The compound showed a hydrophilic profile (LogP = −3.32 ± 0.14) and 40–60% serum protein binding. Clinically, [^68^Ga]Ga-FAPi-46 PET/CT provided superior lesion delineation compared to [^18^F]FDG, revealing additional mediastinal, supraclavicular, and brain metastases. **Conclusions:** [^68^Ga]Ga-FAPi-46 can be reliably synthesized using automated procedures under routine radiopharmacy conditions, meeting regulatory quality standards and demonstrating excellent stability. Its enhanced lesion detectability compared with [^18^F]FDG in the first patient case supports its potential value for oncological staging and clinical implementation.

## 1. Introduction

Since its introduction in 1976, the radiolabeled glucose analogue fluorodeoxyglucose labelled with fluorine-18 ([^18^F]FDG) has been widely used in nuclear medicine, establishing itself as the most utilized positron emission tomography (PET) radiopharmaceutical. Initially prominent in oncologic imaging, its application has progressively expanded to neurological studies, infection imaging, and the assessment of myocardial viability [1]. However, several limitations have become evident over time. These include reduced sensitivity in detecting well-differentiated tumors or specific histological subtypes with low glucose avidity, suboptimal detection in regions with high physiological glucose uptake, and the necessity of a cyclotron for its production [2].

One of the main drivers behind the expanding use of gallium-68 (^68^Ga) in nuclear medicine has been its ability to decouple PET imaging from dependence on costly and centralized cyclotron infrastructure. This achievement, made possible by the development of generator-based systems, allowed the on-site production and availability of PET radiopharmaceuticals [3]. Nonetheless, the translation of ^68^Ga-labeled tracers from basic research to clinical practice has taken several years, mainly due to logistical challenges associated with its relatively short physical half-life (T_1_/_2_ = 67.71 min) and the time required for the commercial development and regulatory approval of ^68^Ga generators [4].

Among the molecular targets recently highlighted in the literature is the Fibroblast Activation Protein (FAP), a membrane-bound serine endopeptidase belonging to the dipeptidyl peptidase-4 (DPP4) family. FAP exhibits enzymatic activity and participates in cell signaling by forming complexes with other proteins, such as integrins—particularly α_3_β_1_ [5]. This glycoprotein is normally absent in the stroma of healthy tissues but is expressed by activated fibroblasts during tissue remodeling and repair. Moreover, FAP expression has been observed in several non-malignant conditions, including wound healing, rheumatoid arthritis, atherosclerotic plaques, and fibrotic diseases [6,7].

The epitope of FAP was identified as a promising molecular target, motivated by the central role of cancer-associated fibroblasts (CAFs) in the tumor microenvironment [8]. CAFs can constitute up to 90% of the total tumor mass in desmoplastic human tumors [9]. Because of their activated phenotype, CAFs constitutively express FAP, providing high target specificity. Building on this biological rationale, the Heidelberg team developed a series of FAP-targeted radiotracers, commonly referred to as FAP compounds [9,10]. These agents exhibit minimal off-target uptake and low background activity, resulting in high-contrast molecular imaging and offering a valuable diagnostic alternative for tumors that are not readily detectable with [^18^F]-FDG [10]. Moreover, since the molecular target resides in the stromal compartment rather than in tumor cells themselves, FAP imaging offers broad applicability across diverse tumor types [11].

Initial strategies to target FAP included monoclonal antibodies [9,12], chimeric antigen receptor T cells, immunoconjugates, peptide-based inhibitors incorporating consensus enzymatic sequences, and experimental vaccines [13]. Subsequent efforts focused on developing small-molecule inhibitors of FAP enzymatic activity, resulting in the synthesis of more than 60 compounds, most of them based on a quinoline scaffold [14].

**Figure 1:**
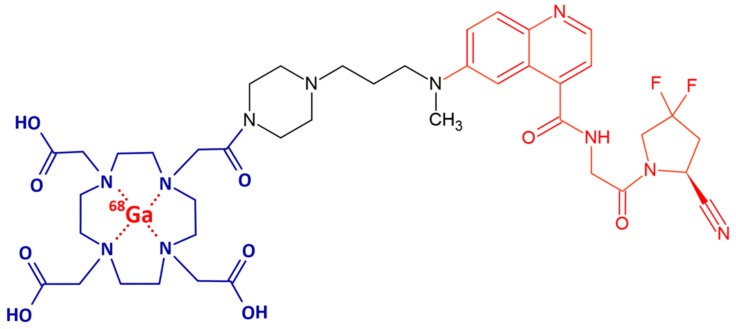
Structural representation of the compound highlighting the DOTA chelator (blue), the molecular spacer (black), and the FAPi (red). IUPAC Name: 2-[4,7-Bis(carboxymethyl)-10-[2-[4-[3-[[4-[[2-(2-cyano-4,4-difluoropyrrolidin-1-yl)-2-oxoethyl]carbamoyl]quinolin-6-yl]-methylamino]propyl]piperazin-1-yl]-2-oxoethyl]-1,4,7,10-tetrazacyclododec-1-yl]acetic acid.

Building on these developments, a series of FAP inhibitor (FAPi) derivatives was conjugated to the bifunctional chelator 1,4,7,10-tetraazacyclododecane-1,4,7,10-tetraacetic acid (DOTA), which is widely used for radiometal coordination and biomolecule functionalization. Among them, FAPi-46 (Figure 1), a potent quinoline-based FAPi, exhibited markedly higher tumor-to-background uptake ratios relative to blood, liver, muscle, and intestine [15]. Clinical translation confirmed its rapid and intense tumor accumulation within minutes post-injection, generating high-contrast PET images and demonstrating its promise for theranostic applications [16].

In 2019, the [^68^Ga]Ga-FAPi-46 radiotracer gained international recognition when the Society of Nuclear Medicine and Molecular Imaging (SNMMI) selected it as the “Image of the Year”. The research team at University Hospital Heidelberg demonstrated its capacity to visualize nearly 30 different cancer types, thereby establishing the foundation for new application in non-invasive diagnosis, tumor staging, and treatment planning [17,18].

Building upon these pioneering findings, our research group has recently implemented [^68^Ga]Ga-FAPi-46 production in a clinical radiopharmacy setting. The present study details the translation process from experimental synthesis to routine clinical use and reports our first patient application of this promising theranostic radiopharmaceutical.

## 2. Results

### 2.1. Radiochemical Purity and Quality Control Results

The syntheses were carried out in-house using an automated synthesis module, with production performed on different days over a six-month period. A total of eight independent batches were produced (*n* = 8), allowing the assessment of inter-batch variability (Table 1). The final product was subjected to standard quality control tests prior to patient administration. To ensure reproducibility and consistency across batches, all productions were performed by a trained and experienced team following standardized operating procedures. The synthesis module and analytical equipment were periodically qualified in accordance with institutional quality control and maintenance protocols.

**Figure 2:**
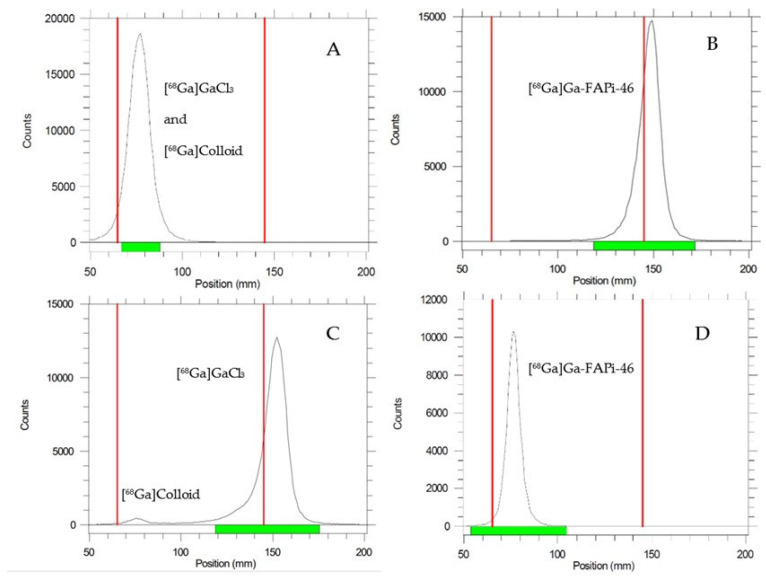
Radiochemical purity analysis using a radio-TLC scanner. (**A**,**B**) Stationary phase: iTLC-SG; mobile phase: 0.1 M ammonium acetate/methanol (1:1, *v*/*v*). (**C**,**D**) Stationary phase: TLC-SG; mobile phase: 0.1 M sodium citrate (pH 5.5).

The radiochemical yield (RCY) was on average greater than 87%, with a clear and colorless final solution. The pH of the final preparation was 4.5. In the evaluation of the radiochemical purity (RCP) of [^68^Ga]Ga-FAPi-46 by ascending chromatography (iTLC-SG in 0.1 M ammonium acetate/methanol—1:1), the radiolabeled compound migrated with solvent front (Rf = 0.9–1.0), allowing quantification of ^68^Ga impurities in the form of free gallium ([^68^Ga]GaCl_3_) and colloidal species, which remained at the strip origin (Rf = 0.0–0.1). For the ascending chromatography system (TLC-SG in 0.1 M sodium citrate), both [^68^Ga]Ga-FAPi-46 and the colloidal species were retained at the origin (Rf = 0.0–0.1), while [^68^Ga]GaCl_3_ migrated with the solvent front (Rf = 0.9–1.0), allowing its quantification separately from the colloidal fraction, as demonstrated in Figure 2.

The results indicated high RCP (98.34 ± 0.82), consistent with the values obtained by solid-phase extraction (SPE) using Sep-Pak C18 cartridges (98.39 ± 0.76). Bacterial endotoxin testing demonstrated levels below 2.5 EU/mL. In conjunction with approved membrane integrity testing, these findings confirmed that the final [^68^Ga]Ga-FAPi-46 preparation complied with sterility and apyrogenicity requirements, ensuring that the radiopharmaceutical was produced under aseptic conditions suitable for safe intravenous administration to patients.

### 2.2. Radiochemical Stability of [^68^Ga]Ga-FAPi-46 in Saline and Human Serum

**Figure 3:**
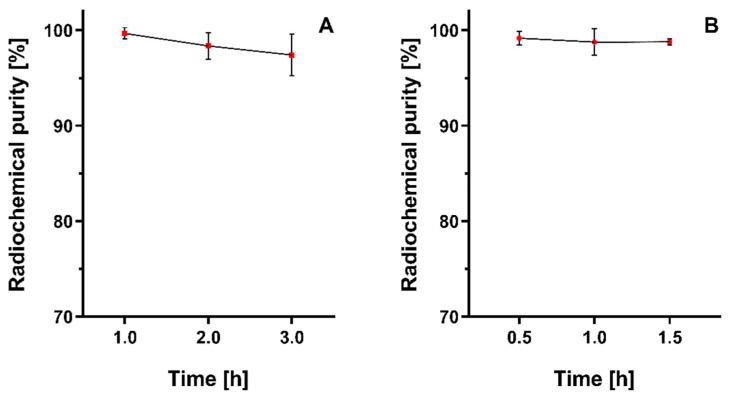
Radiochemical stability assessment of [^68^Ga]Ga-FAPi-46 in (**A**) saline solution (room temperature) and (**B**) human serum (37 °C), assessed through ascending chromatography. Data are expressed as ‘mean ± SD’ (*n* = 3–4). No statistically significant difference was observed within the time interval for the radiochemical stability in saline solution (*p* = 0.1758) and human serum (*p* = 0.8388). ANOVA was conducted, followed by Tukey’s multiple comparisons test.

**Figure 4:**
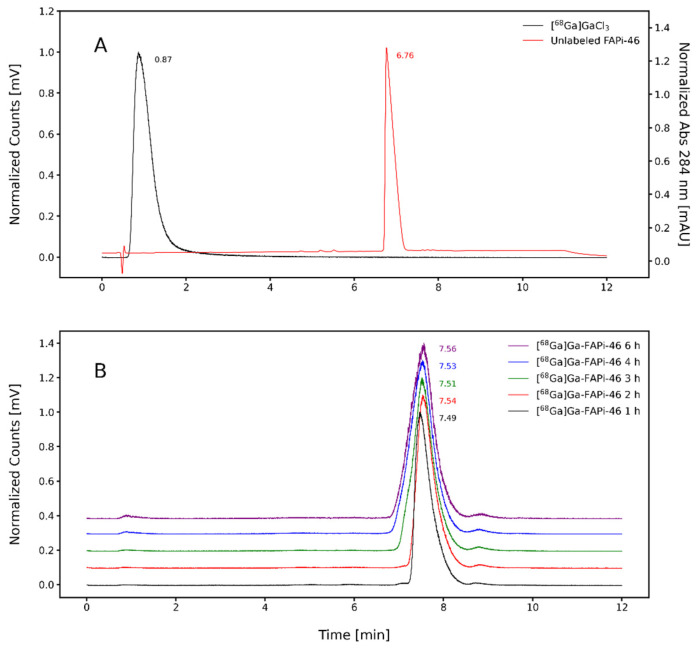
RP-HPLC chromatographic profiles: (**A**) Free [^68^Ga]GaCl_3_ and the unlabeled FAPi-46 precursor; (**B**) [^68^Ga]Ga-FAPi-46 evaluated at different time points after radiolabeling (radiochemical stability).

The radiochemical stability of [^68^Ga]Ga-FAPi-46 was assessed in saline (0.9% NaCl) and human serum using the above-mentioned ascending chromatography systems at 1, 2, and 3 h and at 30, 60, and 90 min post-labeling, respectively (Figure 3). Additionally, radiochemical stability in saline was also investigated by RP-HPLC up to 6 h post-labeling. Chromatographic profiles present the [^68^Ga]GaCl_3_ (Rt = 0.8 min) and unlabeled FAPi-46 (Rt = 6.7 min), as illustrated in Figure 4. Both analytical techniques confirmed high radiochemical stability of [^68^Ga]Ga-FAPi-46, with RCP consistently exceeding 95%.

### 2.3. In Vitro Characterization

The partition coefficient (P) of [^68^Ga]Ga-FAPi-46 was determined, the logarithm of P (Log P) was calculated, and the result was −3.32 ± 0.14 (*n* = 10), confirming the hydrophilic characteristics of the compound. In addition, serum protein binding was also determined after 60 min of incubation at 37 °C, yielding values from 40 to 60% in three different samples.

### 2.4. Case Report

A 50-year-old female patient with a recent diagnosis of pulmonary adenocarcinoma was referred for initial oncologic staging. Chest computed tomography revealed a solid pulmonary nodule measuring 1.9 cm in the medial segment of the middle lobe, along with enlarged mediastinal lymph nodes, suggestive of nodal involvement. Additionally, brain magnetic resonance imaging demonstrated a nodular lesion in the left cingulate gyrus. Whole-body PET/CT imaging was performed using a Biograph mCT scanner (Siemens Medical Solutions, Germany), 60 min after intravenous administration of 185 MBq of [^68^Ga]Ga-FAPi-46, with an acquisition time of 4 min per bed position.

**Figure 5:**
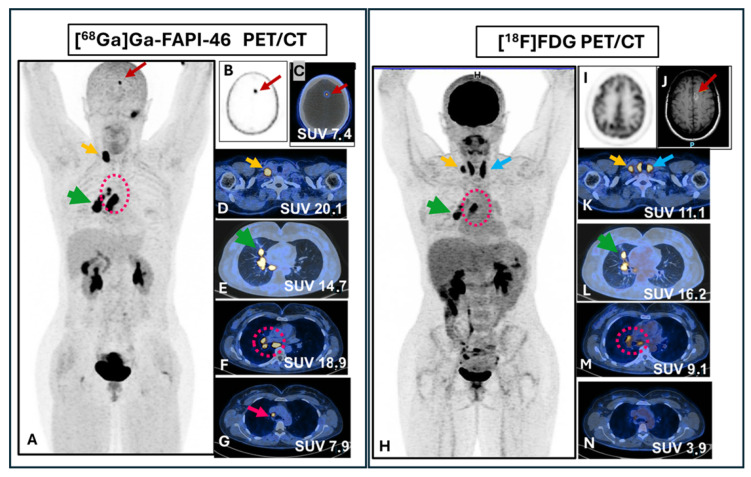
[^68^Ga]Ga-FAPi-46 and [^18^F]FDG PET/CT of a 50-year-old woman with lung adenocarcinoma for initial staging. (**A**–**G**): [^68^Ga]Ga-FAPi-46 PET/CT. (**H**–**N**): [^18^F]FDG PET/CT. (**A**,**H**): Maximum intensity projection (anterior view). (**D**–**G**): [^68^Ga]Ga-FAPi-46 PET/CT and (**K**–**N**): [^18^F]FDG PET/CT axial fusion images. PET/CT shows intense uptake of both (**E**) [^68^Ga]Ga-FAPi-46 and (**L**) [^18^F]FDG in the primary tumor in the right lung (green arrows). Tracer uptake is also observed in ipsilateral pulmonary hilum and subcarinal lymph nodes metastases (pink circles and arrows) and in a right supraclavicular lymph node metastasis (yellow arrow). Of note, PET/CT with [^68^Ga]Ga-FAPi-46 showed greater degree of uptake in mediastinal and supraclavicular lymph nodes (**F**,**G**,**D**) in comparison to [^18^F]FDG (**M**,**N**,**K**). A nodular lesion in central nervous system shown on head MRI ((**J**), red arrow) is shown on PET/CT with [^68^Ga]Ga-FAPi-46 ((**B**,**C**), red arrows) but is not identified on [^18^F]FDG PET/CT (**I**). Thyroid uptake due to thyroiditis (blue arrow) is observed in [^18^F]FDG PET/CT (**K**) but not in [^68^Ga]Ga-FAPi-46 images (**D**).

The patient subsequently underwent both [^18^F]FDG and [^68^Ga]Ga-FAPi-46 PET imaging (Figure 5). Intense radiotracer uptake was demonstrated in the primary pulmonary lesion on both [^18^F]FDG and [^68^Ga]Ga-FAPi-46 PET/CT. However, [^68^Ga]Ga-FAPi-46 PET/CT provided clearer delineation of the primary tumor and revealed additional mediastinal and supraclavicular lymph node metastases compared with [^18^F]FDG PET/CT. Moreover, [^68^Ga]Ga-FAPi-46 PET/CT showed superior detectability of a brain metastasis.

## 3. Discussion

The development of fibroblast activation protein inhibitors has followed a stepwise development strategy to improve tumor retention while preserving favorable clearance from non-target tissues. In a comparative study, Loktev et al. evaluated several quinoline-based derivatives, reporting FAPI-04 as an early clinically applicable tracer characterized by rapid tumor uptake but relatively fast washout, which prompted further structural optimization. Subsequent compounds such as FAPI-21 and FAPI-35 demonstrated improved tumor retention; however, these modifications were associated with increased uptake in non-target organs (e.g., liver and muscle), resulting in less favorable tumor-to-background ratios. Within this series, FAPI-46 emerged as a well-balanced tracer, combining tumor uptake comparable to FAPI-21 with significantly improved tumor-to-organ ratios in preclinical models and clinical imaging. In contrast, FAPI-55 exhibited prolonged tumor retention and higher absolute uptake, features that favor therapeutic applications but may be suboptimal for routine diagnostic imaging due to extended residence time in non-target tissues [15].

More comprehensive analyses, as summarized by Czuczejko et al. (2026) [19] in recent reviews of radiolabeled FAPI radiotracers, further reinforce this distinction between ligands with a diagnostic or therapeutic focus. In particular, peptide-based compounds such as FAPI-2286 exhibit very high binding affinity and prolonged tumor retention, supporting their primary development for radionuclide therapy rather than short-interval diagnostic PET imaging. Within this framework, FAPI-46 represents an optimized balance between pharmacokinetic performance and clinical practicality. Importantly, this balanced biological behavior is well aligned with labeling using short-lived radionuclides such as gallium-68, as it favors rapid target accumulation, fast clearance from non-target tissues, and timely image acquisition. This rationale underpins the choice of FAPI-46 in the present study and provides the basis for the automated radiolabeling and quality control strategy described below [19].

In our study, after standardizing the automated synthesis process of [^68^Ga]Ga-FAPi-46, the results obtained for the assays performed and their respective acceptance criteria were in compliance with the current national health rules, as well as with monographs for other ^68^Ga-based radiopharmaceuticals and recent reports in scientific literature [3]. Automated productions performed using the synthesis module yielded highly reproducible results under controlled conditions, ensuring compliance with good practices and maintaining low levels of operator radiation exposure [3,20].

Radiolabeling efficiency data demonstrated that the compound was obtained with a high radiochemical yield of approximately 90% and a final product radiochemical purity exceeding 98%, corroborating the recently published results by Brusa et al. (2025) [21]. Both analytical methods employed for the determination of radiochemical purity produced comparable results; however, the Sep-Pak C18 evaluation proved to be a faster and more practical alternative, given the short half-life of gallium-68 and the need for rapid product qualification prior to dispensing, since it does not require chromatographic development.

The analyzed compound demonstrated high and consistent stability in both saline and human serum, with no evidence of degradation observed at any of the evaluated time points. Radiochemical stability data obtained in saline confirmed that radiolabeling remained stable for up to 6 h, supporting its potential use in routine nuclear medicine protocols. However, administration is typically performed at earlier time points due to the short half-life of the radionuclide and the required activity for image acquisition. The stability study conducted in human serum confirmed that the radiolabeled compound remained stable at 60 min—the time frame commonly adopted in clinical practice—and maintained its stability up to 90 min, thus allowing for the acquisition of delayed images.

The in vitro characterization of the compound demonstrated that [^68^Ga]Ga-FAPi-46 exhibits a hydrophilic character (LogP = −3.3), consistent with other FAPi ligands reported in the literature, such as [^68^Ga]Ga-FAPi-04 (logD7.4 = −2.4) and [^18^F]FGlc-FAPi (logD 7.4 = −1.0). However, the serum protein binding fraction observed for [^68^Ga]Ga-FAPi-46 was comparable to that reported for [^18^F]FGlc-FAPi (45% binding) yet distinct from that for [^68^Ga]Ga-FAPi-04 (10%) [22].

The clinical case reported in this work is consistent with emerging evidence supporting the promising role of [^68^Ga]Ga-FAPi-46 PET/CT in oncologic assessments, offering improved lesion detection compared with conventional [^18^F]FDG PET/CT.

In this context, a voxel-level digital biopsy approach combined with network analysis and clustering has been used to characterize kinetic signatures of healthy tissue, non-malignant pathological tissue, and malignant lesions. By analyzing the kinetics of [^68^Ga]Ga-FAPi-46 across organs, this strategy enabled the identification of distinct clusters and kinetic features, which may facilitate lesion characterization and diagnosis. Overall, these findings are consistent with our clinical observations, supporting the potential of [^68^Ga]Ga-FAPi-46 to provide complementary biological information beyond conventional imaging [23,24,25].

Along similar lines, clinical studies conducted by Kuper et al., 2025 [26] further support the relevance of [^68^Ga]Ga-FAPi-46 in oncologic imaging. In patients with triple-negative breast cancer, [^68^Ga]Ga-FAPI-46 PET has shown detection rates comparable to [^18^F]FDG PET while providing complementary biological information. Moreover, the high tumor uptake observed in a subset of patients with advanced disease highlights the theranostic potential of this radiotracer, which is in agreement with the clinical applicability observed in our study [26].

In addition to biological performance, methodological aspects such as acquisition timing have also been investigated for [^68^Ga]Ga-FAPi-46 PET imaging. Hoppner et al., 2023 [27] demonstrated that in patients with suspected recurrence of pancreatic ductal adenocarcinoma, early (20 min p.i.) and late (60 min p.i.) acquisitions yield similar detection rates for malignant and non-malignant lesions, indicating that early imaging is feasible. Nevertheless, dual-timepoint imaging may provide additional value for differential diagnosis in selected cases, supporting flexible acquisition protocols for unclear FAP-positive findings [27,28].

Therefore, [^68^Ga]Ga-FAPi-46 PET/CT may contribute to more accurate initial staging and ultimately influence clinical management and outcomes in lung cancer and other solid malignancies. Within this broader clinical context, recent advances have further elucidated the in vivo behavior of [^68^Ga]Ga-FAPi-46 in different tissues, strengthening its role in clinical molecular imaging.

## 4. Materials and Methods

### 4.1. Radiolabeling of FAPi-46 with ^68^Ga

**Figure 6:**
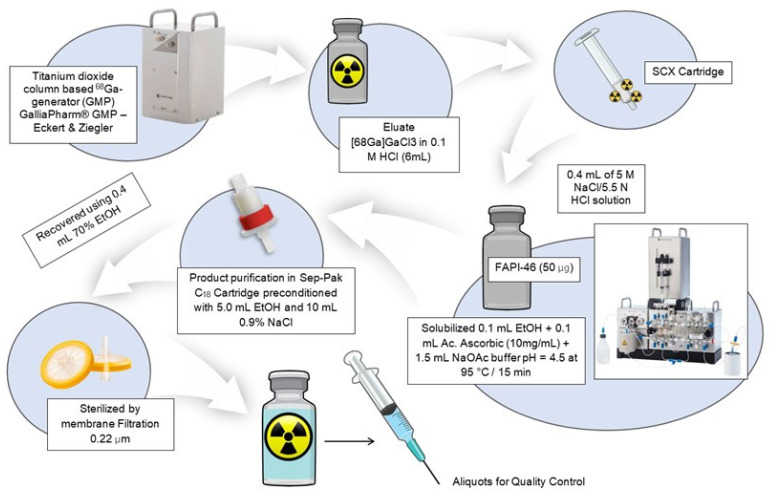
Schematic representation of the automated synthesis and purification process for [^68^Ga]Ga-FAPi-46. Adapted from NIAID NIH BioArt Source [29].

The precursor FAPi-46 was obtained from MedChemExpress (Monmouth Junction, NJ, USA), and radiolabeling was performed using an automated synthesis module (Lab PharmTracer, Eckert & Ziegler, Berlin, Germany) (Figure 6). [^68^Ga]GaCl_3_ was eluted from a GMP-compliant generator (GalliaPharm-Eckert & Ziegler, Berlin, Germany) with 6 mL of 0.1 M HCl and trapped on a strong cation-exchange (SCX) cartridge. The radionuclide was subsequently eluted with 0.5 mL of a 5 M NaCl/5.5 N HCl solution (95:5, *v*/*v*) into a reaction vial containing 50 μg of FAPi-46 dissolved in 1.5 mL of acetate buffer (pH 4.5), 0.1 mL of ethanol, and 0.1 mL of ascorbic acid (10 mg/mL). The reaction mixture was heated at 95 °C for 15 min. The crude product was purified using a C18 cartridge (Sep-Pak Light, Waters, Eschborn, Germany), preconditioned with 5.0 mL of ethanol followed by 10.0 mL of 0.9% saline. The product was subsequently eluted with 0.4 mL of 70% ethanol in water and passed through a 0.22 μm sterile filter (Cathivex-GV, Millipore, Darmstadt, Germany). The final formulation was diluted with 6 mL of 0.9% saline.

### 4.2. Radiochemical Yield

Radiochemical yield was calculated as the ratio of the activity measured in the final product to the total activity initially loaded onto the synthesis module (including waste, cation-exchange cartridge, reaction vial, membrane filter, and Sep-Pak C18).

### 4.3. Radiochemical Quality Control

Radiochemical purity was evaluated using three chromatographic methods: (i) Ascending chromatography was performed both on instant thin-layer chromatography silica gel (iTLC-SG) plate with 1.0 M ammonium acetate/methanol (1:1, *v*/*v*) as the mobile phase and thin-layer chromatography silica gel (TLC-SG) plate with 0.1 M sodium citrate (pH 5.5) as the mobile phase. Radioactivity distribution along the plate was analyzed with a radio-TLC imaging scanner (AR-2000, Eckert & Ziegler, Berlin, Germany/WinScan 3.14. 2013), and the retention factor (Rf) values for [^68^Ga]Ga-FAPi-46 and ^68^Ga-impurities were determined; (ii) Solid-phase extraction (SPE) was carried out using preconditioned Sep-Pak C18 Classic cartridges, with ethanol and saline; (iii) RP-HPLC analyses, an ultra HPLC system (1290 Infinity II UHPLC-Agilent Technologies, United States/OpenLab CDS 2.15.26) equipped with a radioactivity detector (Flow-count base B-FC-1000 Model 106—Eckert and Ziegler, Germany) and using an analytical C18 column (100 × 3.0 mm, 2.6 μm) maintained at 25 °C, with mobile phases A (0.1% trifluoroacetic acid in water) and B (0.1% trifluoroacetic acid in acetonitrile). The gradient was initiated at 5% B, with a linear increase in the acetonitrile proportion to 15% from 0 to 10 min. Subsequently, from 10 to 11 min, the system was linearly returned to 5% B and maintained under this condition until 12 min. The flow rate was set at 1.0 mL/min, and UV detection was carried out at 214 nm.

### 4.4. Radiochemical Stability in Saline

The radiochemical stability of [^68^Ga]Ga-FAPi-46 was assessed in saline (0.9% NaCl) at room temperature (*n* = 3). Briefly, at 60, 120, and 180 min after synthesis, aliquots were analyzed by ascending chromatography and RP-HPLC, as described in Section 4.3.

### 4.5. Radiochemical Stability in Human Serum

The radiochemical stability of [^68^Ga]Ga-FAPi-46 was also assessed in human serum (*n* = 3). Briefly, 50 µL of the radiopharmaceutical (corresponding to 30 µCi—1,1 MBq) was added to 100 µL of human serum, and the mixture was incubated at 37 °C under gentle agitation (500 rpm). At 30, 60, and 120 min, aliquots of 40 µL were collected and subjected to protein precipitation by adding 100 µL of cold acetonitrile. After centrifugation, the supernatant was analysed by ascending chromatography, as described in Section 4.3.

### 4.6. Partition Coefficient Determination

The partition coefficient (P) of [^68^Ga] Ga-FAPi-46 was determined using a biphasic system consisting of n-octanol (500 µL) and 0.9% saline (475 µL) (*n* = 5). A 25 µL aliquot of [^68^Ga]Ga-FAPi-46 was added to the solvent mixture, vortexed for 1 min, and allowed to equilibrate for 5 min. The samples were then centrifuged at 14,000 rpm for 5 min. Subsequently, 100 µL aliquots were carefully collected from both the organic and aqueous phases, and their radioactivities were measured using a Wizard2™ 3” 2480 automatic gamma counter (Wizard^2^ 2480-0010). The partition coefficient (P) was calculated as the ratio of the activity in the organic phase to that in the aqueous phase, and the logarithmic value (Log P) was then derived.

### 4.7. Serum Protein Binding Determination by Ultrafiltration

To assess the extent of radiopharmaceutical binding to serum proteins, two experimental groups were prepared. In the test group, 150 µL of [^68^Ga]Ga-FAPi-46 was added to 1350 µL of human serum. In the control group, the same volume of radiopharmaceutical was added to 1350 µL of sterile saline. Both mixtures were homogenized and incubated at 37 °C for 60 min. Following incubation, six aliquots (250 µL each) were collected from the test group and two aliquots of the same volume from the control group. Each aliquot was transferred to Amicon^®^ ultrafiltration tubes (10,000 Da MWCO) and centrifuged at 14,500 rpm for 5 min using a miniSpin Plus centrifuge (Eppendorf, Hamburg, Germany). The radioactivity in the retentate (protein-bound and/or nonspecifically retained fraction) and the filtrate (free fraction) was quantified with a Wizard2™ 3″ 2480 automatic gamma counter (PerkinElmer, Waltham, MA, USA). Data from the control group were used to estimate nonspecific retention, which was subtracted from test group values to calculate the percentage of specific serum protein binding according to the following equation:

$$\%Protein\;Binding=\frac{{(Retained}_{Test}-{Nonspecific}_{Control})\times 100}{{Retained}_{Test}+{Filtered}_{Test}}$$

### 4.8. Case Report

A 50-year-old female patient with a recent diagnosis of pulmonary adenocarcinoma underwent a [^68^Ga]Ga-FAPi-46 (185 MBq) PET/CT. Whole-body PET/CT examinations (Biograph mCT, Siemens Medical Solutions, Erlangen, Germany) were acquired 60 min after administration using an acquisition time of 4 min per bed position. Images were reconstructed using an iterative reconstruction algorithm. Informed consent was obtained from this patient to use the PET/CT images for research purposes.

### 4.9. Statistical Analysis

Statistical analysis was performed using GraphPad Prism v8.0.2 software (GraphPad Software Inc.—La Joya, CA, USA. Quantitative results were expressed as mean ± SD, and means were compared using one-way ANOVA followed by Tukey’s post hoc test to detect multiple group differences. A threshold of *p* < 0.05 was considered statistically significant.

## 5. Conclusions

This study demonstrated that [^68^Ga]Ga-FAPi-46 can be reliably produced by in-house automated synthesis at a hospital radiopharmacy with high radiochemical yields, excellent purity, and adequate physicochemical properties for clinical application. The compound exhibited good stability in conditions simulating routine nuclear medicine practice and showed in vitro characteristics consistent with other FAP-targeted radiopharmaceuticals currently under investigation. In addition, the clinical imaging results illustrated its capability for improved lesion detection when compared with [^18^F]FDG PET/CT, supporting its diagnostic value in oncology. Together, these findings indicate that [^68^Ga]Ga-FAPi-46 is a promising radiopharmaceutical for PET imaging and may contribute to advances in cancer detection and staging.

## Figures and Tables

**Table 1:** Summarizes the results obtained from the synthesis of [^68^Ga]Ga-FAPi-46 across the different production days.

Test	Release Criteria	Results
Radiochemical Yield [%]	N/A	87.87 ± 1.29
Appearance	Clear, Colorless	Clear, Colorless
pH	4.0–8.0	4.5
Radiochemical Purity (TLC) [%]	>95	98.34 ± 0.82
Radiochemical Purity (Sep-Pak C18) [%]	>95	98.39 ± 0.76
Endotoxin Analysis [EU/mL]	<175	<2.5
Membrane Integrity [bar]	≥3.4	Approved
Volume [mL]	N/A	6.0

The results were expressed as mean ± standard deviation (SD), when applicable (*n*= 8).

## Data Availability

The original contributions presented in this study are included in the article. Further inquiries can be directed to the corresponding author.
